# A novel and efficient method to induce allospecific CD8^+^ memory T lymphocytes

**DOI:** 10.1002/jcla.23972

**Published:** 2021-08-31

**Authors:** Lei Yang, Qingyun Huang, Jianping Fu, Zhimin Lin, Qiqi Mao, Lili Zhao, Xingxin Gao, Songlin Chen, Guangzong Hua, Sheng Li

**Affiliations:** ^1^ The First Affiliated Hospital of Guangxi University of Chinese Medicine Nanning China; ^2^ Guangxi University of Chinese Medicine Nanning China; ^3^ Ningbo Medical Center Lihuili Hospital Ningbo China; ^4^ The General Hospital of the Armed Police Force of Guangxi Nanning China; ^5^ The First Affiliated Hospital of Guangxi University Nanning China

**Keywords:** allogeneic skin transplantation, CD8^+^ memory T cells, intraperitoneal injection

## Abstract

The aim of the current study was to establish a simple method for effectively inducing memory T lymphocytes by the intraperitoneal injection of spleen lymphocytes into mice. In total, 75 mice were divided into the following groups: an injection group administered three doses of spleen lymphocytes (1 × 10^6^, 5 × 10^6^, and 1 × 10^7^ cells), a transplantation group in which a 0.25‐cm^2^ skin section from C57BL/6 mice was transplanted onto the back of the recipient, and a control group in which an equal volume of phosphate‐buffered saline was injected. At 1, 2, or 3 months following transplantation, the following parameters were evaluated: quantity of T lymphocytes, percentage of cluster of differentiation 8^+^ (CD8^+^) memory T cells, and proliferation index of purified CD8^+^ memory T cells. No significant differences among groups were detected at 1 month (*p* > .05). However, the injection group administered 1 × 10^6^ cells exhibited the highest proportion of CD8^+^ memory T cells among all groups at 2 months, and the proportions of CD8^+^ T cells were higher in the three injection groups than in the skin transplantation and control groups at 3 months. The proportions of memory T cells were higher in the injection groups administered 5 × 10^6^ or 1 × 10^7^ cells than in the skin transplantation and control groups at 3 months. The newly established method effectively induces memory T lymphocytes via the intraperitoneal injection of spleen lymphocytes *in vivo* and has potential applications in the field of immunotherapy.

## Graphical abstract

Processing of donor samples: The details of the memory T‐cell induction strategy are shown in Figure 1. All mice underwent aseptic surgery. The donor C57BL/6 mice were sacrificed by neck dislocation, and the skin grafts were cut into sections of 0.25 cm^2^ followed by disinfection by soaking in 75% alcohol for 5 min. The spleens of donor mice were aseptically excised and used to make lymphocyte suspensions. The lymphocytes were counted using a hemocytometer, diluted to 1 × 10^6^, 5 × 10^6^, and 1 × 10^7^, resuspended in 0.2 ml of phosphate‐buffered saline (PBS), and used for intraperitoneal injections. Skin transplantation and injections: The mice in the skin transplantation group were anesthetized using 0.1 ml/10 g 1% sodium pentobarbital following systemic disinfection. A 0.25‐cm^2^ section of skin on the back of each mouse was replaced with the donor skin and sutured around the edge of the section. Mice in the injection groups were injected with three doses (1 × 10^6^, 5 × 10^6^, or 1 × 10^7^ cells) of the prepared lymphocyte suspensions, taking care to avoid puncturing the internal organs, and the same volume of PBS was injected into control mice. Following surgery, mice in the skin transplantation group were disinfected with 75% alcohol daily to avoid infection. The donor skin that was sutured onto the back of the recipients became necrotic and was shed ~8 days postoperation, with gradual wound healing. Collection of T lymphocytes: After the mice were sacrificed by cervical dislocation, they were placed in the right lateral position, the skin surface was disinfected with 75% alcohol, the skin was cut with sterile surgical instruments, and the spleen was removed. The spleen was placed in a vessel containing PBS, and cells in the spleen were carefully collected with syringes and fully suspended in PBS. An autoclaved filter was used to filter the cell suspension. Centrifugation was performed and the supernatant was discarded. Red cells in the pellet were lysed by the addition of red blood cells lysate buffer. Cells were washed with PBS three times and centrifuged to obtain PBMCs, which were ready for subsequent experiments.

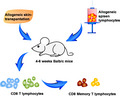

## 1 INTRODUCTION

Memory T cells, consisting of effector memory T cells (T_EM_) and central memory T cells (T_CM_), are necessary for protective immunity against invading pathogens, particularly under immunosuppression.^1^, ^2^ Adaptive immune responses depend on immunocytes, which recognize and eliminate recurrent pathogens, resulting in the generation of memory lymphocytes. The capacity of memory T cells to rapidly mobilize and initiate a potent recall response enhances protective immunity against previously encountered pathogens. However, the low activation thresholds and the susceptibility of memory T cells to conventional immunosuppressive agents are significant obstacles to successful transplantation.^3^, ^4^, ^5^

A number of functions have been described for cluster of differentiation 8^+^ (CD8^+^) T cells, particularly for CD44^+^CD62L^−^CD8^+^ memory T cells in certain clinical situations,^6^ including autoimmunity, organ transplantation, and cancer. CD8^+^ memory T cells serve essential roles in these conditions, contributing to a poor prognosis.^7^, ^8^ In the late immune response, the majority of effector T cells undergo apoptosis, except for a few T cells with long‐term survival, forming memory cells. When the same antigen stimulates the body again, the immune memory is triggered to provide protection. Long‐term, effective immune protection depends on the functions and quantity of memory T cells. The quantity of memory T cells under normal conditions is very low and cannot meet the needs of scientific research, Therefore, the acquisition of CD8^+^ memory T cells is an indispensable and very important aspect, and an effective induction method is urgently needed.^9^

The traditional method for inducing CD8^+^ memory T cells by allogeneic skin transplantation.^10^, ^11^ At the same time, some scholars claim that allogeneic skin transplantation can produce memory T lymphocytes, due to transplant rejection.^12^, ^13^ In contrast, the new method in this study is based on the principle that allogeneic cell transplantation produces acute rejection.^14^, ^15^ So, the aims of the present study were to establish a novel and efficient method to induce CD8^+^ memory T cells and to compare this new approach with the traditional method, that is, to compare their ratios and proliferative capacity.

## 2 MATERIALS AND METHODS

### 2.1 Mice

A total of 75 female BALB/c mice (4–6 weeks old) were divided into the following five groups: three injection groups receiving different doses of cells (1 × 10^6^, 5 × 10^6^, or 1 × 10^7^), a skin‐transplantation group, and a control group. The mice were followed up for a period of 3 months, during which they underwent allogeneic skin transplantation and the intraperitoneal injection of spleen lymphocytes (C57BL/6 mice).

C57BL/6 mice were obtained from Vital River Laboratories Co., Ltd., and BALB/c mice were provided by the Guangxi Medical University Laboratory Animal Centre (Nanning, China). The mice were housed in the Guangxi Medical University Laboratory Animal Centre in a pathogen‐free environment with standard temperature and humidity conditions. All animal experiments were performed in accordance with the Federation of European Laboratory Animal Science Association guidelines, and the protocols were approved by the Animal Ethics Committee of Guangxi Medical University of Chinese Medicine (Nanning, China).

### 2.2 Processing of donor samples

**FIGURE 1:**
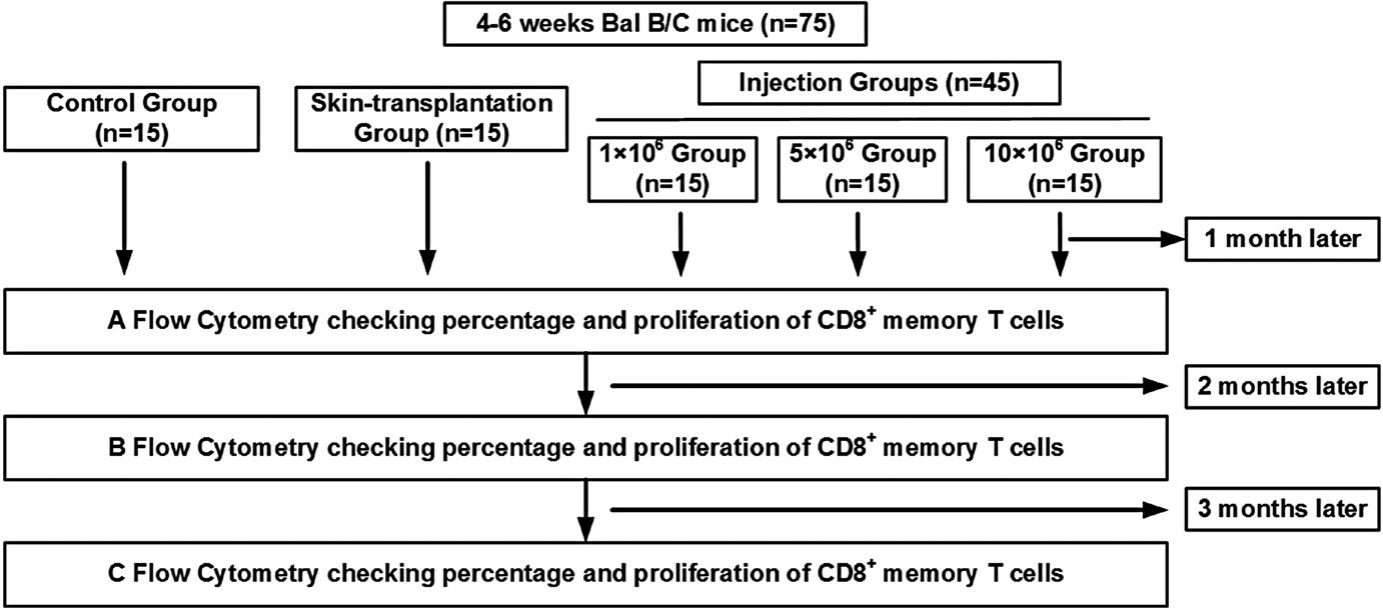
Technical route. Flowchart illustrating the detection of items in each group

The details of the memory T‐cell induction strategy are shown in Figure 1. All mice underwent aseptic surgery. The donor C57BL/6 mice were sacrificed by neck dislocation, and the skin grafts were cut into sections of 0.25 cm^2^ followed by disinfection by soaking in 75% alcohol for 5 min. The spleens of donor mice were aseptically excised and used to make lymphocyte suspensions. The lymphocytes were counted using a hemocytometer, diluted to 1 × 10^6^, 5 × 10^6^, and 1 × 10^7^, resuspended in 0.2 ml of phosphate‐buffered saline (PBS), and used for intraperitoneal injections.

### 2.3 Skin transplantation and injections

The mice in the skin transplantation group were anesthetized using 0.1 ml/10 g 1% sodium pentobarbital following systemic disinfection. A 0.25‐cm^2^ section of skin on the back of each mouse was replaced with the donor skin and sutured around the edge of the section. Mice in the injection groups were injected with three doses (1 × 10^6^, 5 × 10^6^, or 1 × 10^7^ cells) of the prepared lymphocyte suspensions, taking care to avoid puncturing the internal organs, and the same volume of PBS was injected into control mice. Following surgery, mice in the skin transplantation group were disinfected with 75% alcohol daily to avoid infection. The donor skin that was sutured onto the back of the recipients became necrotic and was shed ~8 days postoperation, with gradual wound healing.

### 2.4 Collection of T lymphocytes

After the mice were sacrificed by cervical dislocation, they were placed in the right lateral position, the skin surface was disinfected with 75% alcohol, the skin was cut with sterile surgical instruments, and the spleen was removed. The spleen was placed in a vessel containing PBS, and cells in the spleen were carefully collected with syringes and fully suspended in PBS. An autoclaved filter was used to filter the cell suspension. Centrifugation was performed and the supernatant was discarded. Red cells in the pellet were lysed by the addition of red blood cells lysate buffer. Cells were washed with PBS three times and centrifuged to obtain PBMCs, which were ready for subsequent experiments.

### 2.5 Purification of T cells

CD8^+^ memory T cells were isolated from purified whole T cells using MACS CD8 Microbeads with the CD62L biotin cocktail^16^ according to the manufacturer’s instructions (BD Biosciences).

### 2.6 Carboxyfluorescein succinimidyl ester labeling and T cell proliferation assay

Purified CD8^+^ memory T cells were washed and resuspended in 1 ml of serum‐free medium with 0.1 μl of carboxyfluorescein succinimidyl ester (CFSE). Following 8 min of incubation at 37°C, the CFSE‐labeled T cells were washed three times with PBS. Spleen lymphocytes from the C57BL/6 mice were processed with mitomycin C for 30 min at 37°C and then co‐cultured with the CFSE‐labeled T cells at a ratio of 1:10 in the absence of human interleukin (IL)‐2 for 3 days. For the induction and proliferation of antigen‐specific T cells, human IL‐2 was added at a low dose of 10 U/ml on day 4. On day 5 or 6, CFSE‐labeled T cells were analyzed by single‐color fluorescence‐activated cell sorting and Modfit 2 T‐cell Cycle Analysis software (Verity Software House).

### 2.7 Statistical analysis

Data are expressed as means ± standard deviation. After testing for homogeneity of variance, the *t*‐test was used for comparisons between two groups. All analyses were performed with SPSS for Windows version 18.0 (SPSS Inc.). A *p*‐value of <.05 was considered to indicate a statistically significant difference.

## 3 RESULTS

### 3.1 Quantity of T lymphocytes at different time points in each group

**FIGURE 2:**
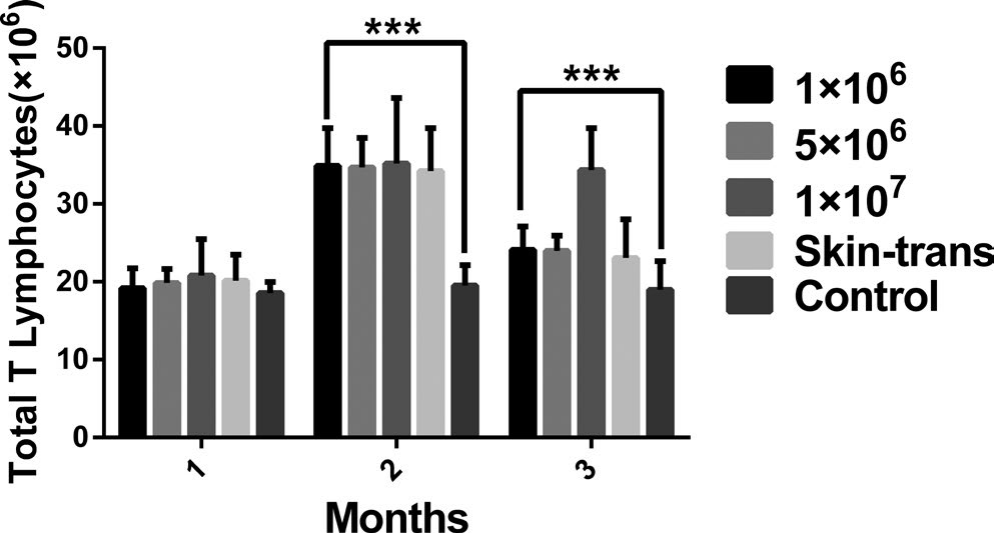
Quantity of total T lymphocytes at the different time points in five groups. ***p < .001. In the 2nd and 3rd months, the quantity of total T lymphocytes in the four experimental groups were significantly higher than that in the control group (*p* < .001)

There were no significant differences between the injection group and the transplantation group, with respect to the quantity of T lymphocytes at different time points. The quantity of T cells started to decline at month 2 in all groups. However, the total numbers of T lymphocytes in the experimental groups were significantly higher than those in the control group at 2 and 3 months (but not at 1 month) (*p* < .05; Figure 2 and Table 1).

**TABLE 1:** Percentage of CD8^+^ memory T cells and total T lymphocytes at different time points

Time	Injection group			Skin‐trans group	Control group
	1 × 10^6^ cells	5 × 10^6^ cells	1 × 10^7^ cells		
Percentage of CD8^+^ T cells (%)					
1st month	15.10 ± 1.972	15.22 ± 2.290	14.44 ± 2.003	14.16 ± 2.031	11.59 ± 4.764
2nd month	41.34 ± 2.806^*,**^	32.46 ± 2.290^*,**^	33.40 ± 0.765^*,**^	28.04 ± 1.318^*^	11.33 ± 4.872
3rd month	19.46 ± 5.063^*,**^	57.20 ± 1.051^*,**^	44.60 ± 12.749^*,**^	37.58 ± 1.919^*^	11.76 ± 4.974
Total T lymphocytes (×10^6^ cells)					
1st month	19.20 ± 2.530	19.84 ± 1.824	20.80 ± 4.665	20.16 ± 3.318	18.56 ± 1.431
2nd month	34.88 ± 4.852^*^	34.67 ± 3.820^*^	35.20 ± 8.390^*^	34.24 ± 5.496^*^	19.52 ± 2.629
3rd month	24.12 ± 2.964^*^	23.98 ± 1.964^*^	24.36 ± 5.351^*^	23.06 ± 4.947^*^	18.96 ± 3.687

Data is presented as means ± standard deviation.

^*^ *p* < .05 vs. the control group; ^**^ *p* < .05 vs. the skin transplantation group.

### 3.2 Alterations in CD8^+^ memory T cells

**FIGURE 3:**
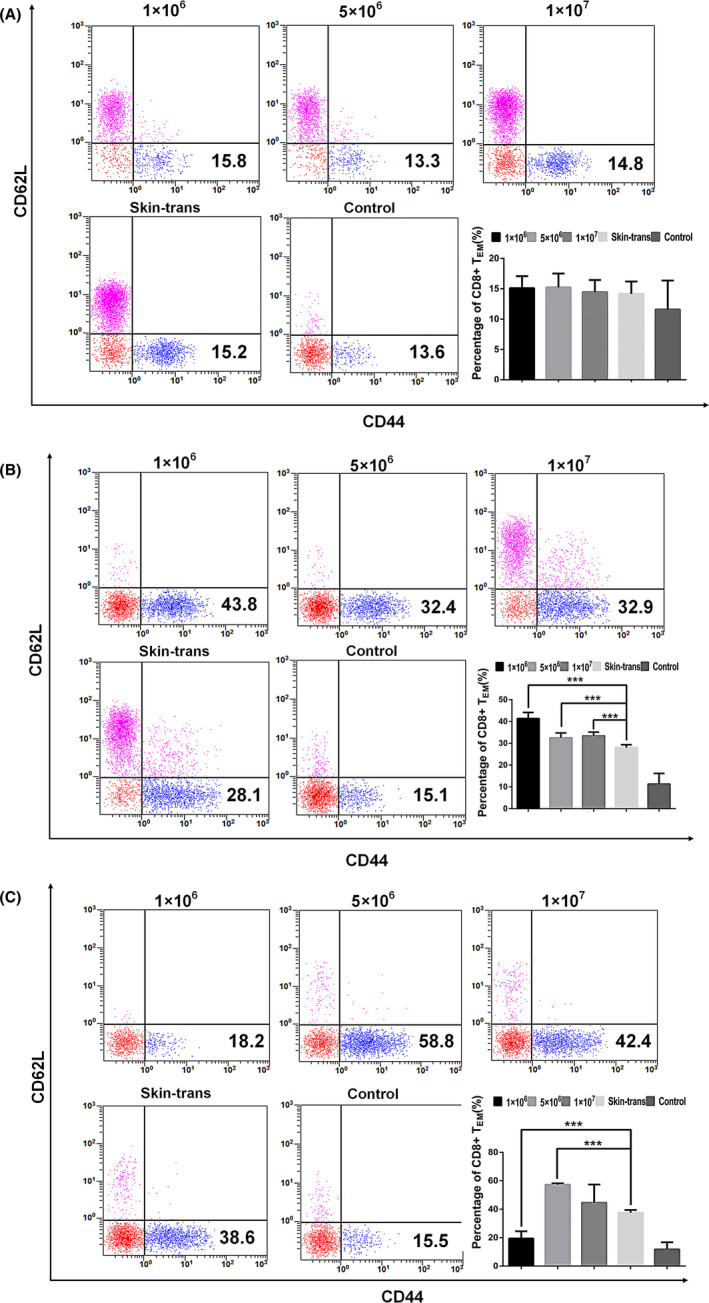
Percentage of CD8^+^ memory T cells in different groups at different time points, as measured by flow cytometry. Dots in the fourth quadrant indicate the CD8^+^ memory T cells, which are CD8^+^CD44^+^CD62L^−^. (A) There were no significant differences in the percentage of CD8^+^ memory T cells at 1‐month posttransplantation (*p* > .05). (B) At 2 months, the injection groups exhibited a higher percentage of CD8^+^ memory T cells than those in the skin transplantation or control groups, with the greatest difference in the low‐dose group (1 × 10^6^ cells). (C) Percentage of CD8^+^ memory T cells at 3 months posttransplantation. **p < .01, ***p < .001. CD, cluster of differentiation

As presented in Figure 3A, there were no significant differences in CD8^+^ memory T cell percentages among groups at 1‐month postoperation (*p* > .05). The percentage of CD8^+^ memory T cells in the skin transplantation group was higher than that in the control group but lower than those in the injection groups at 2 months postoperation (*p* < .001). Among the injection groups, the percentage of CD8^+^ T cells was the highest in the low‐dose group (*p* < .001; Figure 3B). At 3 months, the percentages of CD8^+^ memory T cells in the experimental groups were significantly higher than that in the control group (*p* < .001), except for the low‐dose group (*p* > .05). Compared with the skin transplantation group, the mid‐dose group had a higher percentage of CD8^+^ memory T cells (*p* < .001); however, the percentage in the mid‐dose group did not differ from that in the high‐dose group (*p* > .05; Figure 3C). The percentage of CD8^+^ T cells and the quantity of total T cells are presented in Table 1. There were no significant differences in the quantity of T lymphocytes among groups at each time point. The number of total T cells was significantly higher at 2 months than at the 1‐ and 3‐month time points (*p* < .05).

### 3.3 Separation efficiency of CD8^+^ memory T cells

**FIGURE 4:**
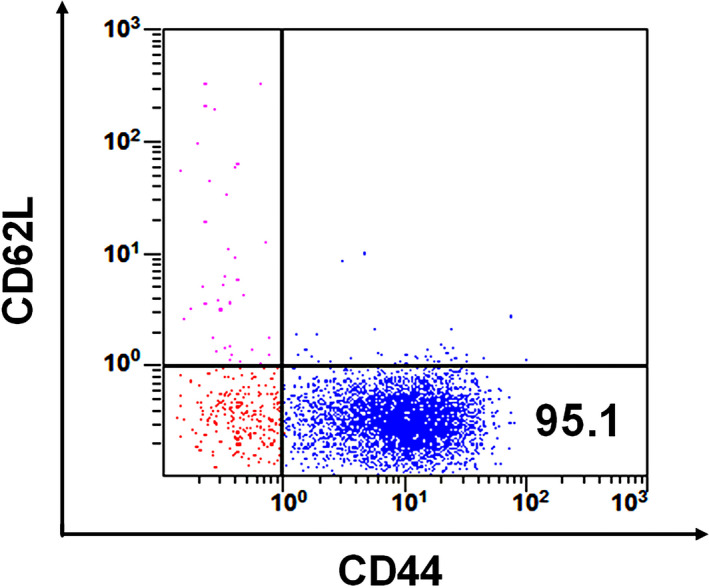
Purification efficiency of CD8^+^ memory T cells. Dots in the fourth quadrant indicate CD8^+^ memory T cells, which are CD8^+^CD44^+^CD62L^−^. The final purity of CD8^+^ memory T cells reached 95.1%, which was sufficient for subsequent experiments experiment

As shown in Figure 4, the CD8^+^ memory cells were separated from the whole spleen lymphocytes with an efficiency of 95.1%. Additionally, the purity of the selected T‐cell populations was >80% in all cases, as assessed by flow cytometry using FACScan (Canto Ⅱ, BD). The purity was sufficient for subsequent experiments.

### 3.4 Proliferation of purified CD8^+^ memory T cells

**FIGURE 5:**
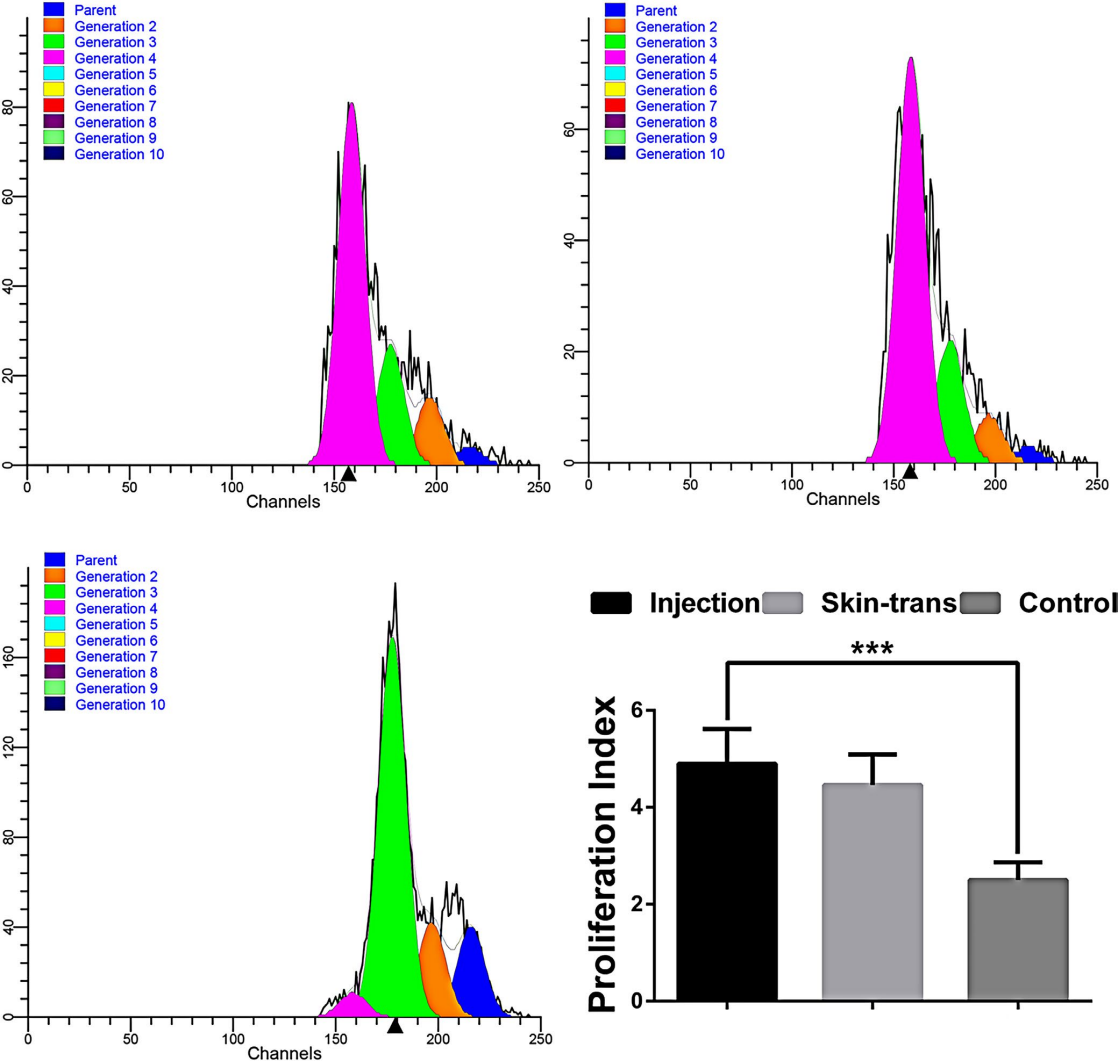
Proliferation of purified CD8^+^ memory T cells. Proliferation of CD8^+^ memory T cells from the experimental (injection and skin transplantation) and control groups; there were no significant differences among experimental groups. Different generations are listed in the upper left corner, marked with different colors. ***p < .001

Following purification, CD8^+^ memory T cells stimulated by allogeneic lymphocytes were detected in the injection groups, skin transplantation group, and control group. As presented in Figure 5, there were no significant differences among the experimental groups; however, the proliferative capacity of T cells in the experimental groups was significantly higher than that in the control group.

## 4 DISCUSSION

Immunological memory, based on specific memory T cells, B cells, and plasma cells following the initial immune response, is a core issue in immunology.^17^ CD8^+^ memory T cells serve a crucial role in transplantation immunity responses. Owing to the ease of preparation and detection, CD8^+^ memory T cells are more widely used than CD4^+^ T cells and B cells for research. The differential expression of CD62L enables memory T cells to be divided into two groups: T_CM_ (CD62L^+^) and T_EM_ (CD62L^−^).^18^, ^19^

Memory T cells play an important role in clinical organ transplantation, once the recipient develops immune memory, memory T cells will be present in the blood, and when the blood flows through the transplanted organ, the memory T cells will rapidly differentiate into cytotoxic T lymphocytes (CD8^+^ T cells). The effective inhibition of the proliferation and biological function of memory T cells is vital for extending the life of transplanted organs.

CD8^+^ memory T‐lymphocytes in the present study were antigen‐specific T‐lymphocytes. Lanzavecchia^20^ observed that memory T cells have a low survival rate and proliferative capacity; therefore, following the initial exposure to the antigen, the quantity of memory T cells declines. In the present study, following induction for 3 months, the immunological memory formed gradually and the number of lymphocytes was reduced, including the memory T cells, despite an increase in the percentage of total T cells. And it was also found that individual samples in the high‐dose group had lower TEM than the medium‐dose group at the third month, but statistical analysis showed no statistical difference (*p* > .05). For the reason of this phenomenon, we analyzed that it might be due to the differentiation of some T cells into TCM at the end of the immune response. Intermediate dose splenocytes cause more severe rejection than the low‐dose group so that large amounts of TEM can still be detected at 3 months.

Long‐term and effective immune protection depends on the capacity and quantity of memory T cells; however, the proliferative ability contributes to the quantity of memory cells. Therefore, after confirming the percentage of the CD8^+^ memory T cells, the proliferation index of the T cells induced by the two methods was compared. CD8^+^ memory T cells purified from whole T cells of the different groups were co‐cultured with allogeneic spleen lymphocytes from C57BL/6 mice to compare the proliferation capacity between the different methods. As CFSE is partitioned equally during cell division,^21^, ^22^, ^23^, ^24^ this technique is commonly used for monitoring T‐cell division and determining the association between the division and differentiation of T cells. In the current study, there was no difference in the proliferation index of CD8^+^ memory T cells isolated from the injection groups and the skin transplantation group.

In conclusion, the two methods for inducing CD8^+^ memory T cells yield satisfactory results; however, the newly developed intraperitoneal injection method is more convenient compared with the traditional skin transplantation method. The proliferation of purified CD8^+^ memory T cells against allogeneic lymphocytes from the two groups was equivalent; however, the induction process mediated by intraperitoneal injection was more rapid. Compared with the complicated sterile surgical procedure required for the skin transplantation method, the intraperitoneal injection of allogeneic lymphocytes is simple and convenient. The present study did not explore the underlying mechanisms, related signaling pathways, or the suppressive role of CD8^+^ memory T‐cells to other antigens. Therefore, further research is needed to reveal the detailed mechanism by which the intraperitoneal injection method induces abundant CD8+ memory T cells.

## CONFLICT OF INTEREST

None.

## Data Availability

The data that support the findings of this study are available from the corresponding author upon reasonable request.
